# Statin treatment after surgical aortic valve replacement for aortic stenosis is associated with better long-term outcome

**DOI:** 10.1093/ejcts/ezae007

**Published:** 2024-01-25

**Authors:** Emily Pan, Susanne J Nielsen, Maya Landenhed-Smith, Charlotta Törngren, Erik Björklund, Emma C Hansson, Anders Jeppsson, Andreas Martinsson

**Affiliations:** University of Turku, Turku, Finland; Department of Surgery, Central Finland Hospital Nova, Jyväskylä, Finland; Department of Molecular and Clinical Medicine, Institute of Medicine, Sahlgrenska Academy, University of Gothenburg, Gothenburg, Sweden; Department of Cardiothoracic Surgery, Sahlgrenska University Hospital, Gothenburg, Sweden; Department of Molecular and Clinical Medicine, Institute of Medicine, Sahlgrenska Academy, University of Gothenburg, Gothenburg, Sweden; Department of Cardiothoracic Surgery, Sahlgrenska University Hospital, Gothenburg, Sweden; Department of Molecular and Clinical Medicine, Institute of Medicine, Sahlgrenska Academy, University of Gothenburg, Gothenburg, Sweden; Department of Cardiothoracic Surgery, Sahlgrenska University Hospital, Gothenburg, Sweden; Department of Molecular and Clinical Medicine, Institute of Medicine, Sahlgrenska Academy, University of Gothenburg, Gothenburg, Sweden; Department of Medicine, Southern Älvborg Hospital, Borås, Sweden; Department of Molecular and Clinical Medicine, Institute of Medicine, Sahlgrenska Academy, University of Gothenburg, Gothenburg, Sweden; Department of Cardiothoracic Surgery, Sahlgrenska University Hospital, Gothenburg, Sweden; Department of Molecular and Clinical Medicine, Institute of Medicine, Sahlgrenska Academy, University of Gothenburg, Gothenburg, Sweden; Department of Cardiothoracic Surgery, Sahlgrenska University Hospital, Gothenburg, Sweden; Department of Molecular and Clinical Medicine, Institute of Medicine, Sahlgrenska Academy, University of Gothenburg, Gothenburg, Sweden; Department of Cardiology, Sahlgrenska University Hospital, Gothenburg, Sweden

**Keywords:** Surgical aortic valve replacement, Statin, Secondary prevention medication, Outcome

## Abstract

**OBJECTIVES:**

The aim of this study was to evaluate the association between statin use after surgical aortic valve replacement for aortic stenosis and long-term risk for major adverse cardiovascular events (MACEs) in a large population-based, nationwide cohort.

**METHODS:**

All patients who underwent isolated surgical aortic valve replacement due to aortic stenosis in Sweden 2006–2020 and survived 6 months after discharge were included. Individual patient data from 5 nationwide registries were merged. Primary outcome is MACE (defined as all-cause mortality, myocardial infarction or stroke). Multivariable Cox regression model adjusted for age, sex, comorbidities, valve type, operation year and secondary prevention medications is used to evaluate the association between time-updated dispense of statins and long-term outcome in the entire study population and in subgroups based on age, sex and comorbidities.

**RESULTS:**

A total of 11 894 patients were included. Statins were dispensed to 49.8% (5918/11894) of patients at baseline, and 51.0% (874/1713) after 10 years. At baseline, 3.6% of patients were dispensed low dose, 69.4% medium dose and 27.0% high-dose statins. After adjustments, ongoing statin treatment was associated with a reduced risk for MACE [adjusted hazard ratio 0.77 (95% confidence interval 0.71–0.83). *P* < 0.001], mainly driven by a reduction in all-cause mortality [adjusted hazard ratio, 0.70 (0.64–0.76)], *P* < 0.001. The results were consistent in all subgroups.

**CONCLUSIONS:**

The results suggest that statin therapy might be beneficial for patients undergoing surgical aortic valve replacement for aortic stenosis. Randomized controlled trials are warranted to establish causality between statin treatment and improved outcome.

**Figure:**
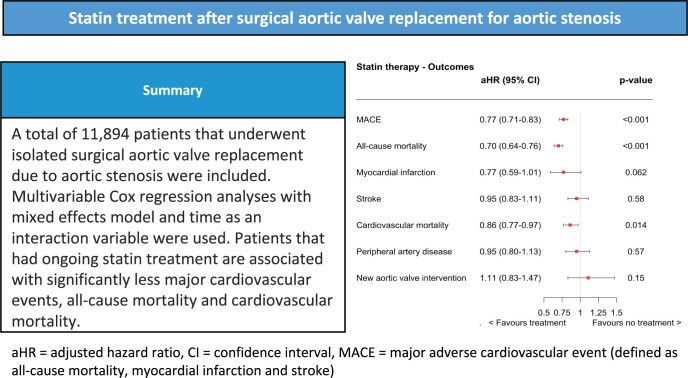

## INTRODUCTION

Aortic valve stenosis due to calcification affects approximately 1–2% of the population with a higher prevalence (3–5%) in patients >75 years [1, 2]. Aortic stenosis is hence the most prevalent valvular heart disease requiring intervention in developed countries and the prevalence is expected to rise with the ageing population [3]. Currently, the only curative treatment for severe aortic stenosis is to replace the diseased valve either surgically [surgical aortic valve replacement (SAVR)] or through a transcatheter-based approach [transcatheter aortic valve implantation (TAVI)].

The pathophysiology of aortic stenosis shares many similarities to coronary artery disease, including progressive calcification, chronic inflammation and common risk factors such as ageing, hyperlipidaemia, male gender, hypertension and smoking [4]. Statins, also known as 3-hydroxy-3-methyl-glutaryl-coenzyme A reductase inhibitors, are effective in reducing cardiovascular events in coronary artery disease patients in both primary and secondary prevention strategies [5, 6]. However, statins have not been shown to halt the disease progression in aortic stenosis patients [7–9], although many of its pleiotropic effects were thought to benefit patients with high cardiovascular burden [10]. Interestingly, studies have shown that statin use is associated with lower mortality and morbidity after the TAVI procedure [11–13]. In contrast, there’s little evidence for statin use after SAVR [14–17].

Our group recently reported that treatment with statins and renin-angiotensin-system inhibitors was associated with a reduced mortality risk in a large cohort of SAVR patients [14]. In the present study, based on partly the same study population, we aimed to investigate other aspects of statin use and long-term outcome after SAVR. More specifically, we investigated in this large, contemporary study cohort: (i) potential associations between statin use after SAVR and major adverse cardiovascular events (MACEs) as well as associations between statin use and all-cause mortality, cardiovascular mortality, myocardial infarction (MI), stroke, peripheral artery disease (PAD), new coronary angiography and new aortic valve intervention, (ii) potential associations between statin use and MACE in predefined subgroups based on age, sex, type of valve prothesis and comorbidities and (iii) potential associations between statin treatment intensity and MACE.

## MATERIALS AND METHODS

### Ethical statement

The study was conducted in accordance with the 1975 Declaration of Helsinki and was approved by the Swedish Ethical Review Authority (registration number 2021-00122, approved 31 March 2021). The need for individual informed consent for this retrospective, registry-based study was waived by the committee.

### Data sources

The data sources have been described previously [18]. The patients were identified in the national Swedish Cardiac Surgery Registry [19] which is a part of the Swedish Web System for Enhancement and Development of Evidence-Based Care in Heart Disease Evaluated According to Recommended Therapies (SWEDEHEART) registry [20]. Information from the SWEDEHEART registry, which has complete coverage of all cardiac surgery procedures in Sweden during the study period [19], was linked to 3 other national registries using the unique identification number that is assigned to each individual in Sweden at birth or upon immigration. Other registries include the National Patient Register, which covers ninth and 10th revision International Classification of Diseases (ICD) codes from all out- and inpatient hospital admissions since 1987, the Cause of Death Registry and the Swedish Prescribed Drug Registry, which covers all dispensed medications since July 2005 based on the Anatomical Therapeutic Classification. The Swedish transcatheter cardiac intervention registry (SWENTRY), which also is a part of SWEDEHEART, was established in 2010 and was used to collect transcatheter aortic valve interventions after SAVR from 2010 and onwards. Supplementary Material, Table S1 displays the ICD-9 and ICD-10 codes used for identification of comorbidities and events, and Anatomical Therapeutic Classification codes used for collecting data on medication, respectively.

### Study population

**Figure 1:**
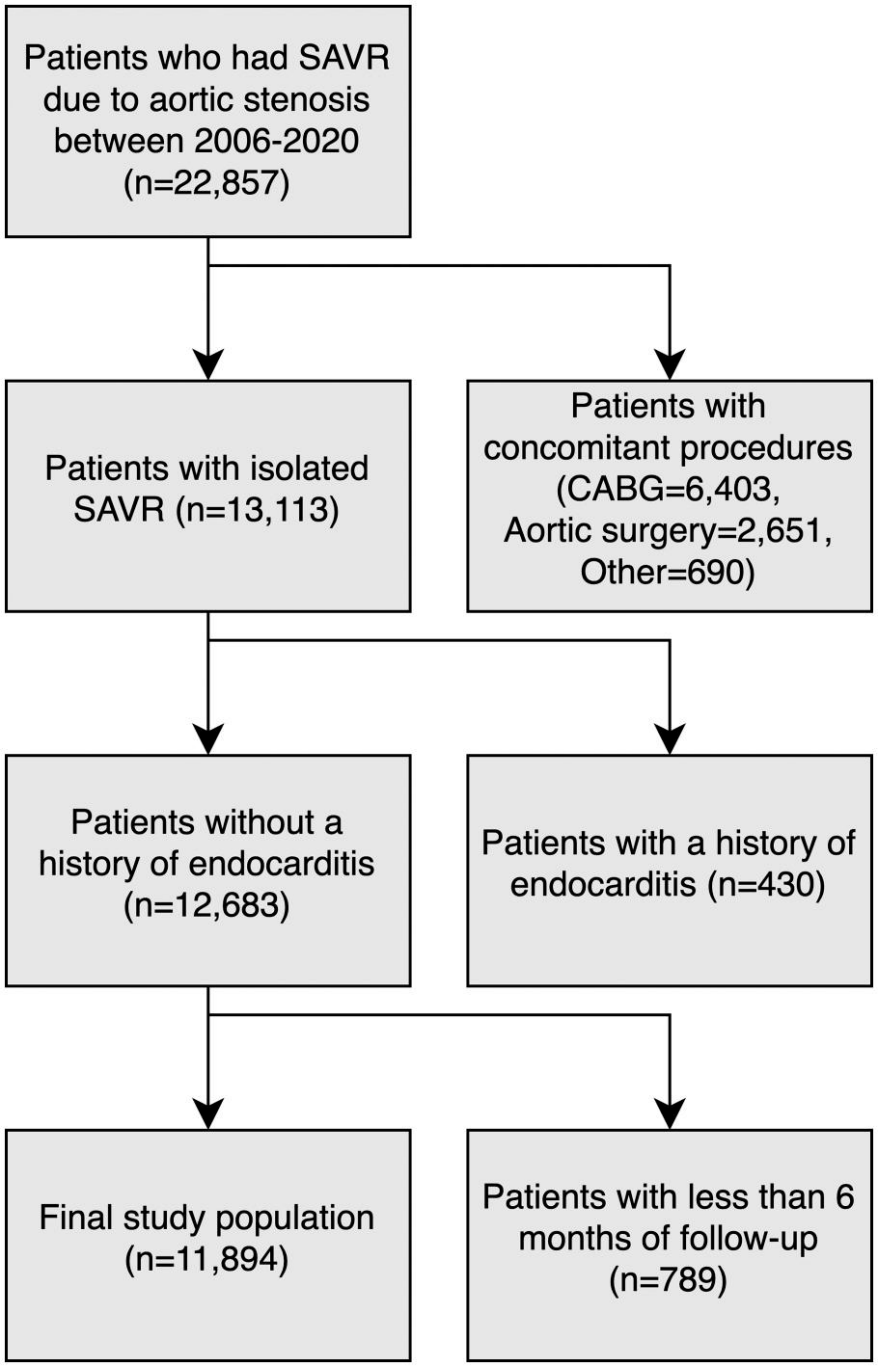
A flowchart illustrating the inclusions and exclusions for patients who underwent SAVR due to aortic stenosis in the final analysis. SAVR: surgical aortic valve replacement.

The study population consists of all consecutive ≥18-year-old patients who underwent their first-time, isolated SAVR surgery due to aortic valve stenosis in Sweden between 1 January 2006 and 31 December 2020. Patients who simultaneously had endocarditis, or underwent any other concomitant surgical procedure, were excluded. Patients who did not complete at least 6 months of follow-up, due to death or emigration, were also excluded from further analysis. The follow-up is complete for all patients until 31 December 2020. A flowchart of patient selection is shown in Fig. 1.

### Definitions

The baseline and the start of follow-up for analysis was set at 6 months after discharge, as we are studying the long-term outcome related to secondary prevention medication. Type and intensity of statin that were dispensed to a patient were defined according to 2018 American College of Cardiology/American Heart Association classification of statin intensity and shown in Supplementary Material, Table S2 [21]. Information about dispensed medications was updated every third month throughout the follow-up time. Two consecutive 3-month periods without registered dispense of medication is considered off-treatment, as previously described [22].

There were 3 variables with missing data: type of prosthesis (*n* = 34, 0.3%), body mass index (*n* = 308, 2.6%) and left ventricle ejection fraction (*n* = 46, 0.4%). Based on the assumption that these data were missing at random, it was handled as a separate category in the analysis.

The primary outcome was MACE, defined as a composite of all-cause mortality, MI or stroke. The secondary outcomes include all-cause mortality, cardiovascular mortality, MI, stroke, PAD and new aortic valve intervention. Heart failure was defined as a clinical diagnosis and includes patients with preserved or reduced left ventricle ejection fraction (</≥50%). Estimated glomerular filtration rate (eGFR) was calculated according to the Chronic Kidney Disease Epidemiology Collaboration formula [23].

The subgroups were defined based on their clinical relevance, prior to the analysis. These include sex, age (≤75 or >75 years), ejection fraction <50%, prior MI, prior stroke, PAD, hypertension, diabetes, hyperlipidaemia, eGFR < or >60 ml/min and type of valve prosthesis. An arbitrary age cut-off of 75 years of age was used for subgroup analysis, based on the guideline directed age recommendation for SAVR and transcutaneous aortic valve intervention.

This manuscript has been written according to recommendations in the Strengthening the Reporting of Observational Studies in Epidemiology (STROBE) statement [24].

### Statistical analysis

Continuous variables are presented as median with interquartile range if not normally distributed. The distributions of the continuous variables were visually inspected using QQ plots. Categorical variables are presented as frequencies with percentages. For baseline comparisons, chi-squared test was used for categorical variables and Student’s *t*-test for continuous variables. The crude incidence rate was calculated by dividing the number of events by cumulative follow-up years and reported as event rates per 100-person years with 95% confidence intervals assuming a Poisson distribution. The cumulative incidence function was used to estimate the occurrence of each type of competing risks as recommended by Austin *et al.* [25]

Variance inflation factor was used to assess collinearity in the model. There were no variables with a high variance inflation factor (all <2.0) indicating that there was no significant collinearity present in the model. All hazard ratios are reported with 95% confidence interval. In the multivariable analysis, Cox proportional hazards regression was complemented with a mixed effects model, using individual’s unique serial number as a random effect. The model was adjusted for age, sex, comorbidities and time-updated use of medications and set prior to data analysis. The following comorbid conditions were adjusted for: hypertension, diabetes, hyperlipidaemia, heart failure, eGFR, prior MI, prior stroke, year of surgery, type of prosthetic valve and ongoing treatment with other secondary prevention medications [renin–angiotensin system (RAS) inhibitors, beta-blockers and platelet inhibitors] to calculate adjusted hazard ratio (aHR). The results from mixed effects model were similar to regular adjusted Cox regression analysis and therefore presented as supplementary material (Supplementary Material, Figs S1 and S2). Due to competing risks, we also used a cause-specific hazards approach for the secondary outcomes that did not include all-cause mortality. The proportional hazards assumption was tested using scaled Schoenfeld residuals. The model did not meet the assumption and therefore, in the main analysis, a time-interaction variable was added to variables that had time-varying effects. In our model, age at operation and year of operation had time-varying effects; therefore, time interaction terms were used for these variables.

The subgroup interaction analysis between ongoing statin use and MACE are illustrated as forest plots and reported as aHR with 95% Cl. The subgroups were analysed separately and in addition to using an interaction term, the *P*-value for interaction is reported.

All tests were two-tailed and interpreted at 0.05 significance level. The statistical analyses were performed using R version 4.2.3. (R: A Language and Environment for Statistical Computing, Vienna, Austria) and RStudio Version 2022.12.0 + 353 (RStudio: Integrated Development for R. RStudio, PBC, Boston, MA, USA).

## RESULTS

### General

Between January 2006 and December 2020, a total of 22 857 patients underwent SAVR due to aortic stenosis in Sweden. After excluding patients that had concomitant procedures, a history of endocarditis or <6 months of follow-up, 11 894 patients were identified and included in the final analysis (Fig. 1). Overall mean age was 69.5 years (standard deviation 11.1), of which 7083 (59.6%) were male and 9369 (79.0%) received a bioprosthetic valve. The median follow-up time was 5.3 years (interquartile range 2.7–8.4, range 0–13.5).

### Baseline characteristics

The characteristics of patients with or without statin at baseline are presented in Table 1. At baseline, 6006 (50.5%) patients were dispensed statin. Patients treated with statin at baseline were significantly older, more often male, had higher body mass index and more comorbidities including hypertension, hyperlipidaemia, atrial fibrillation, renal failure, previous MI and stroke. They were also more often dispensed other secondary prevention medications such as RAS inhibitors and beta-blockers.

**Table 1:** Characteristics of patients undergoing isolated surgical aortic valve replacement due to aortic stenosis according to statin use at baseline

	Patients on statin at baseline (*n* = 6006)	Patient not on statin at baseline (*n* = 5888)	*P*-value
Age (years)	71.1 (SD 8.7)	67.7 (SD 12.8)	<0.001
Female (%)	2332 (38.7%)	2489 (42.3%)	<0.001
Left ventricular ejection fraction <50%	1192 (19.8%)	1148 (19.5%)	0.89
BMI	27.4 (IQR 21.4–33.4)	26.3 (IQR 22.4–33.3)	<0.001
eGFR	74.9 (SD 29.2)	79.4 (29.2)	<0.001
Previous MI	755 (12.6%)	192 (3.3%)	<0.001
Atrial fibrillation	2694 (44.9%)	2448 (41.6%)	<0.001
Heart failure	655 (10.9%)	646 (11.0%)	0.91
Diabetes	1589 (26.5%)	596 (10.1%)	<0.001
Renal failure	377 (6.3%)	277 (4.7%)	<0.001
Previous stroke	288 (4.8%)	135 (2.3%)	<0.001
Hypertension	4420 (73.6%)	3065 (52.1%)	<0.001
Peripheral vascular disease	1061 (17.7%)	1178 (20.0%)	0.001
Hyperlipidaemia	2949 (49.1%)	527 (9.0%)	<0.001
Valve type			
Mechanical prosthesis	1004 (16.8%)	1487 (25.3%)	<0.001
Biological prosthesis	4983 (83.2%)	4386 (74.7%)	<0.001
Other cardiovascular medication			
RAS inhibitor	3638 (60.6%)	2739 (46.5 %)	<0.001
Beta blocker	5044 (84.0%)	4566 (77.5%)	<0.001
ASA	3197 (53.2%)	2445 (41.5%)	<0.001

Values are represented as mean and standard deviation (SD), median and interquartile range (IQR) or number (percentage).

ASA: acetylsalicylic acid; BMI: body mass index; eGFR: estimated glomerular filtration rate; IQR: interquartile range; RAS: renin–angiotensin system; SD: standard deviation.

### Primary outcome

**Figure 2:**
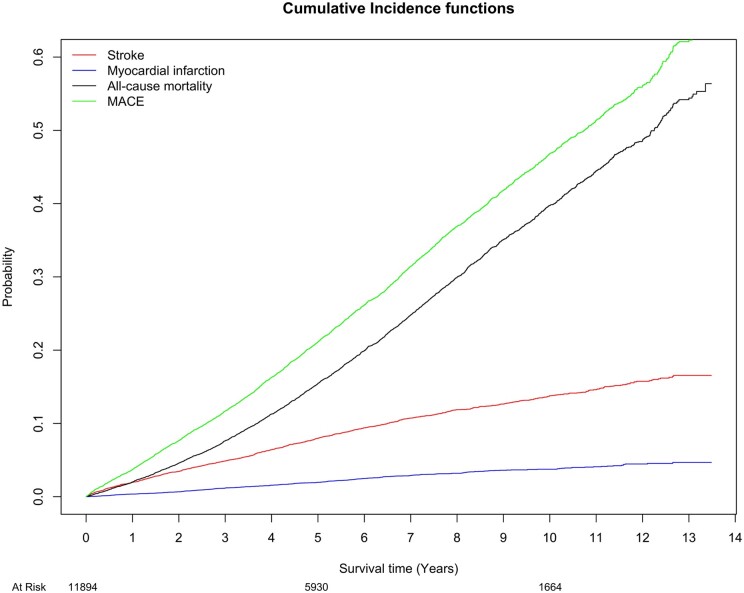
Cumulative incidence function illustrating the probability for stroke, myocardial infarction, all-cause mortality and MACE over time after SAVR for aortic stenosis. MACE: major adverse cardiovascular event; SAVR: surgical aortic valve replacement.

**Figure 3:**
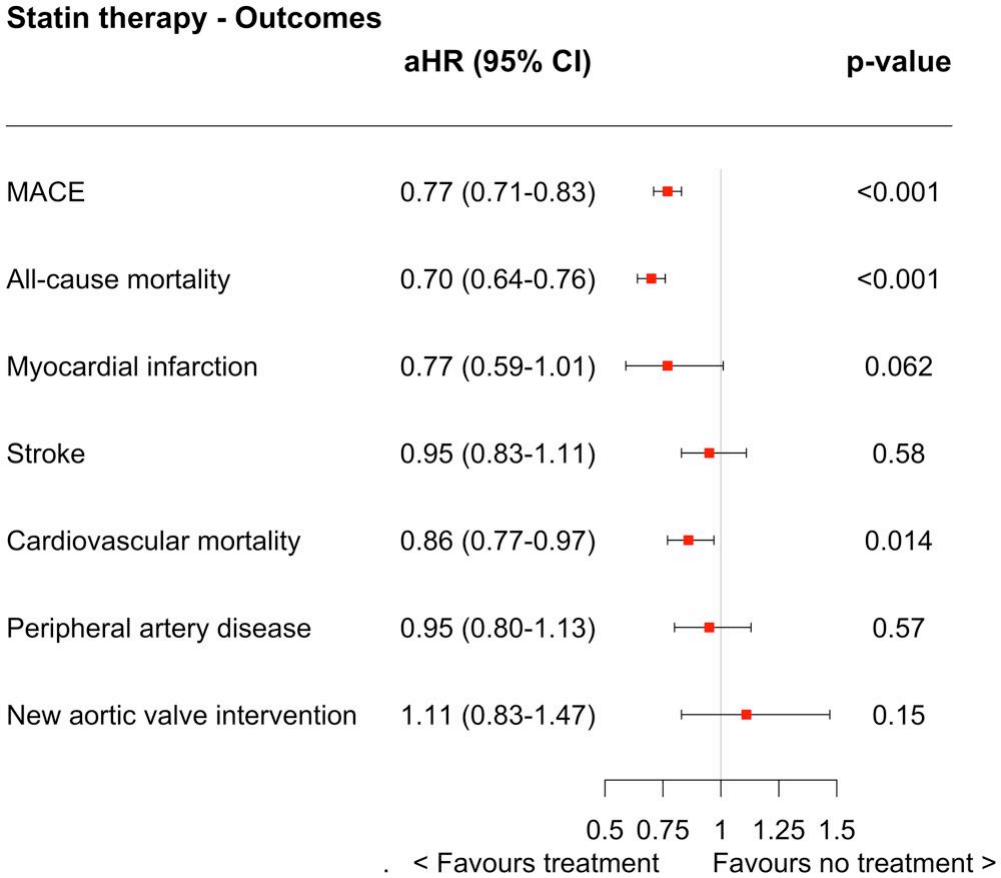
Time-updated Cox regression analysis between ongoing statin use and primary and secondary outcomes illustrated as forest plots and presented as adjusted hazard ratio (aHR) with 95% confidence interval (CI). MACE: major adverse cardiovascular event. The aHR is estimated using Cox regression model that has been adjusted for age, sex, comorbidities, year of surgery, type of prosthetic valve and ongoing treatment of other secondary prevention medication. MACE is defined as all-cause mortality, stroke and myocardial infarction.

During the follow-up, a total of 3666 (30.8%) patients experienced MACE and 2979 (25.0%) died. The cumulative incidence for MACE was 3.7%, 21.1% and 46.8% at 1, 5 and 10 years, respectively (Fig. 2). The crude event rate for MACE per 100-person years was 6.8 (6.5–7.1) in patients dispensed statin at baseline, and 5.7 (5.5–6.0) with those without statin therapy at baseline (Supplementary Material, Table S2). In the adjusted time-updated Cox regression model, ongoing statin therapy was associated with significantly reduced MACE (aHR 0.77 [0.71–0.83], *P* < 0.001) (Fig. 3).

### Secondary outcomes

Figure 2 also illustrates the cumulative incidence for all-cause mortality, MI and stroke. The cumulative incidence for all-cause mortality was 2.0%, 15.4% and 39.7% at 1, 5 and 10 years, respectively. Patients with statin therapy at baseline had slightly higher unadjusted crude event rate for all secondary end-points (Supplementary Material, Table S2). In the adjusted Cox regression model, ongoing statin therapy was associated with significantly reduced risk for all-cause mortality (aHR 0.70 [0.64–0.76], *P* < 0.001) and cardiovascular mortality (0.86 [0.77—0.97], *P* = 0.014), however, no significant associations were found for MI, stroke, PAD or new aortic valve intervention (Fig. 3). Cox regression analysis with mixed effect model showed in addition an association with significantly reduced risk for MI (0.90 [0.83—0.99], *P* = 0.033) (Supplementary Material, Fig. S2).

### Subgroup analyses

**Figure 4:**
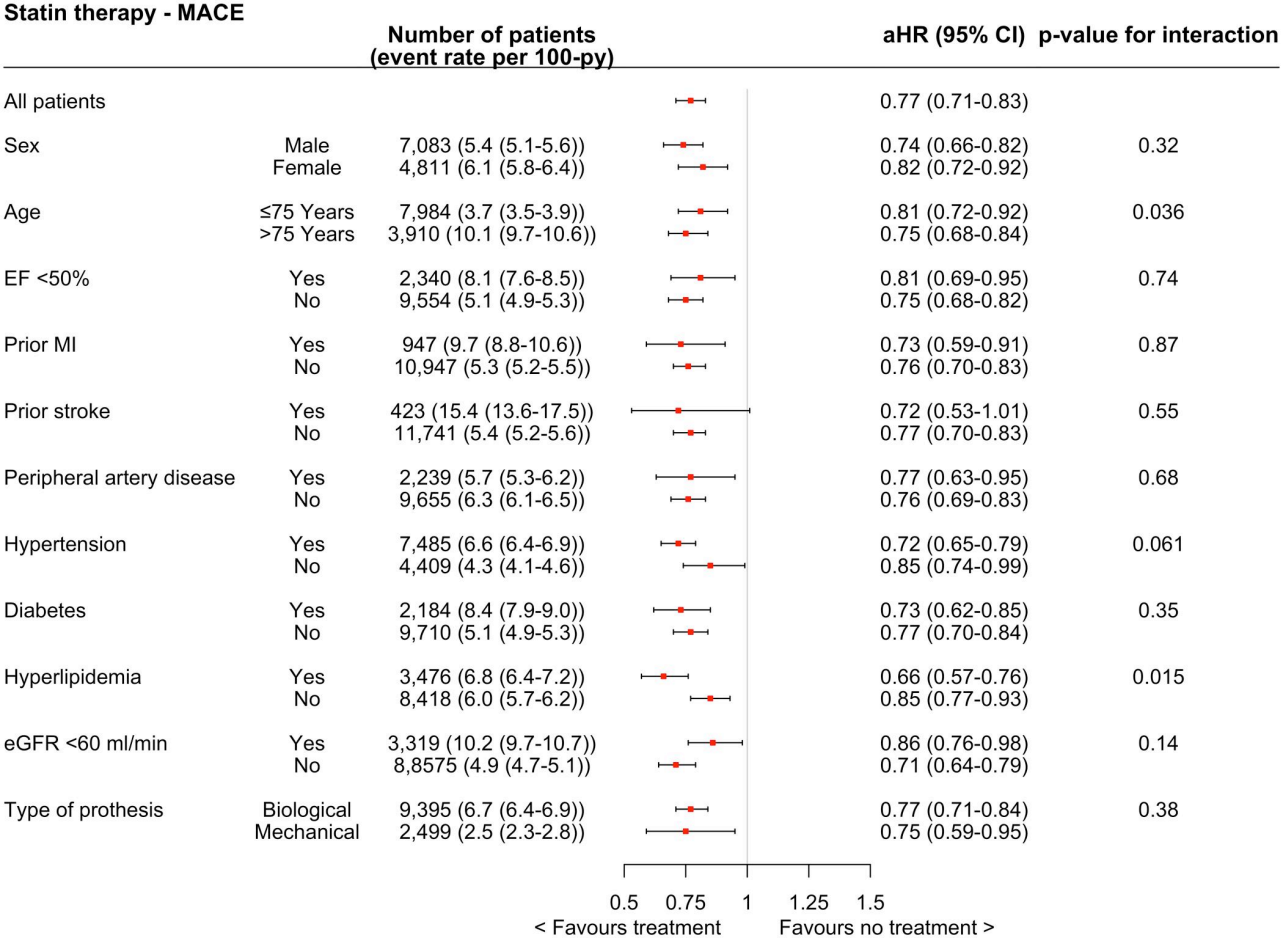
The association between ongoing statin use and predefined subgroups illustrated as forest plots and presented as event rate per 100 patient-year and adjusted hazard ratio (aHR) with 95% confidence interval (CI). eGFR: estimated glomerular filtration rate; EF: ejection fraction; MACE: major adverse cardiovascular event; MI: myocardial infarction.

The benefit associated with statin use was consistent in all predefined subgroups (Fig. 4). Patients above 75 years old or had hyperlipidaemia had even stronger associations (greater risk reduction of events) for MACE when compared to younger patients (interaction *P*-value 0.036) or no hyperlipidaemia (interaction *P*-value 0.015). There were no significant interactions between any other subgroup (all interaction *P*-values > 0.05).

### Statin dispense and intensity comparisons

**Figure 5:**
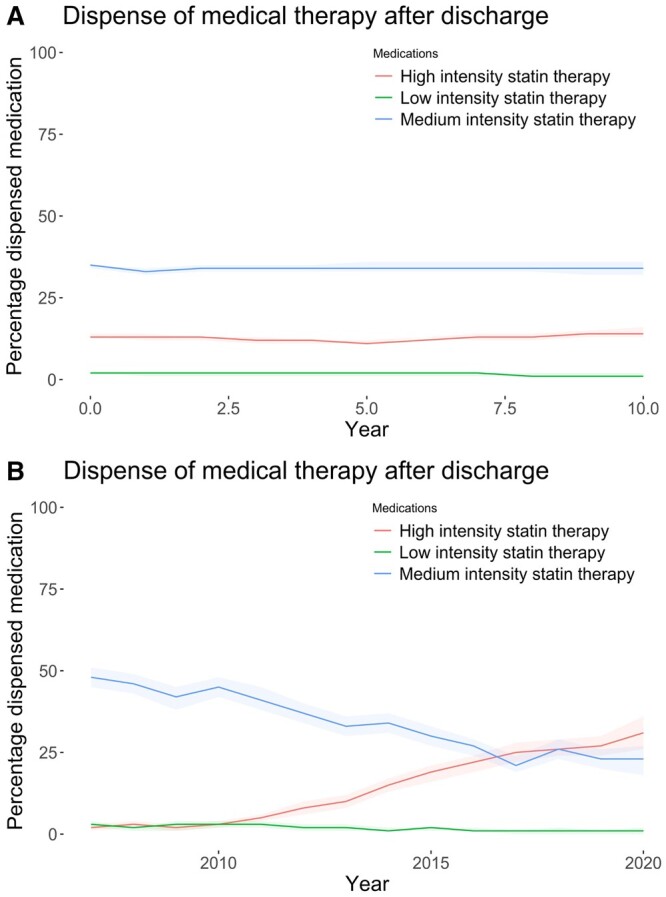
(**A**) Line plots illustrating time-updated statin dispensation over time, stratified by intensity. (**B**) Line plots illustrating time-updated statin dispensation at discharge by year of surgery, stratified by intensity. The shaded areas denote 95% confidence intervals.

Among patients dispensed statins at baseline, 1600 (27.0%) had high, 4107 (69.4%) intermediate and 211 (3.6%) low intensity of statin treatment (Fig. 5A). The overall statin dispense was stable from 5918/11894 (49.8%) at baseline to 3286/6914 (47.5%) at 5 years and 1097/2174 (50.5%) at 10 years with no significant changes within statin intensities. Nevertheless, patients were increasingly dispensed high-intensity statin therapy at discharge after year 2011, eventually surpassing the amount of medium-intensity dispense in 2016 (Fig. 5B).

Ongoing use of medium-intensity statins (aHR 0.74 (0.68–0.80), *P* < 0.001) and high-intensity statins (aHR 0.86 (0.77–0.97), *P* = 0.011) but not low-intensity statins (aHR 0.83 [0.65–1.05], *P* = 0.12), was associated with significantly reduced risk for MACE, when compared to no statin use.

## DISCUSSION

There are 3 main findings in this large, population-based real-world study: (i) ongoing statin use was associated with a significantly reduced risk for MACE in patients that had undergone SAVR due to aortic stenosis, (ii) statin treatment was associated with a reduced risk for MACE in all predefined subgroups and (iii) medium- and high-intensity statin treatment were both associated with a reduced risk for MACE.

SAVR in patients with aortic stenosis remains one of the most common open cardiac procedures performed worldwide and the patients have a substantial life expectancy after the procedure [18]. Therefore, optimizing postoperative treatment strategies to achieve improved outcomes holds significant importance. In the present study, statin use was associated with reduced risks for MACE, all-cause mortality and cardiovascular mortality after SAVR. Three previous observational studies have investigated the associations between statin treatment and outcome after SAVR [14, 15, 17]. In a propensity score-matched analysis with a short follow-up, Angeloni *et al.* [15] reported significant associations between statin treatment and reduced risks for hospital mortality, postoperative cardiac arrhythmias and stroke after isolated heart valve operations. In another larger propensity-matched study, Pullan *et al.* [17] showed that statin therapy was associated with better long-term survival after isolated SAVR, however, a further sub-analysis demonstrated that this applied only to patients who received biological prosthesis. The present study differs from these 2 studies on several important aspects; it is markedly larger and with longer follow-up, it included only patients with SAVR due to aortic stenosis and used time-updated information about statin dispense while the previous studies only included information at baseline. The latter aspect is particularly important, since dispense in individual patients may vary significantly over time. The third previous study by Baranowska *et al.* [14], which partially utilized the same study population as the current study and incorporated time-updated information regarding statin use, found that ongoing treatment with statins and RAS inhibitors was associated with better long-term survival after SAVR. Further evidence for a significant association between statin treatment and better outcome in patients undergoing valve replacement comes from similar studies in patients undergoing TAVI [11–13] and from a study in patients undergoing SAVR due to aortic regurgitation [26].

Statins have several important properties in addition to their lipid-lowering effects, which may explain the associations with improved outcome after SAVR. Statins have been shown to have anti-inflammatory, antithrombotic and antiproliferative properties. Furthermore, statins stabilize atherosclerotic plaques and improve endothelial function [16, 27]. All these effects, also known as pleiotropic effects, may be beneficial for SAVR patients. Once the aortic valve has been replaced, the long-term morbidity and mortality in aortic stenosis patients are thought to be driven by other comorbidities [16, 28]. Interestingly, the benefit of ongoing statin use was mainly driven by all-cause and cardiovascular mortality while there were no significant associations with reduced risks for new MI, stroke or PAD after SAVR. We speculate that this is because a combination of plaque stabilization and systemic pleiotropic properties that could potentially benefit not only patients with baseline comorbidities but also those who might be in higher risk developing cardiovascular diseases and have overall more comorbidities. This is also supported by our subgroup analysis, where both patients with and without a hyperlipidaemia diagnosis seem to benefit from statin use.

Calcified valves are known to have not only ongoing dystrophic calcification but even more active ossification, bone remodelling, macrophage and lymphocyte accumulations [29]. No clinical study has been able to demonstrate that lowering blood cholesterol level with statins slows down calcification and stenosis progression in patients with advanced native aortic valve stenosis [7–9], despite that hyperlipidaemia itself may increase the risk for aortic valve calcification [4, 28]. However, the trials that did not show any effect of statins to thwart the progressive calcification in the aortic valve only included patients that already had heavily calcified valves (aortic valve area 1.01–1.56 cm^2^) and the follow-up time (2–4 years) might have been too short to show effects [7–9]. One may speculate that lipid-lowering treatment could reduce the progression from early valvular inflammation, while deterioration to later disease stages where calcific disease mechanisms are more important, a mechanism which could potentially be beneficial after implantation of a biological prosthesis. Our data, however, did not show an association between statin use and new aortic valve intervention. It should be emphasized that the data does not include indication for intervention, and the average time to a new intervention (5.8 years) is less than what is expected to be caused by restenosis. Furthermore, the number of new aortic intervention was very low (2.4%) and could not be reliably analysed whether there’s a difference between patients receiving biological or mechanical SAVR and statin use. Nevertheless, the use of statins as a secondary prevention strategy in patients undergoing SAVR needs to be investigated in prospective studies.

Almost half (49%) of patients were dispensed with statin at baseline, and the proportion remained stable at ∼50% during follow-up. Interestingly, patients were increasingly dispensed with high-intensity statins, growing from 2% to 27% in 10 years, indicating a shift towards a more aggressive lipid-lowering strategy in recent years. Both medium and high intensity of statins were independently associated with a reduced MACE. Very few patients were dispensed with low-intensity statin, which may explain the absence of a statistically significant association for low intensity with reduced MACE, given that the point estimate was similar between high, medium and low statin intensities.

The current European Society of Cardiology and European Association for Cardio-Thoracic Surgery (ESC/EACTS) guidelines on valvular heart disease [30] do not provide any recommendations on medications after aortic valve replacement in patients with aortic stenosis outside from treating concomitant comorbidities. This reflects the absence of prospective randomized studies that could have shown that medications can alter the prognosis after aortic valve replacement. However, the mounting evidence from observational studies argues for the initiation of prospective studies on statin treatment after SAVR.

### Strengths and limitations

The strengths of this current study include merging and utilizing multiple validated national registries to provide a large, contemporary, population-based, real-world dataset. In addition, the data has a complete follow-up for up to 13.5 years. Every 3-month updated medication data provides a robust basis for analysis and provides confidence that statin dispense is correct during follow-up.

Nevertheless, there are several limitations that should be mentioned. First, we do not have data on blood cholesterol level which would most likely affect the dispensed statin intensity. Second, we do not have the indications for statin dispense in most patients, nor information about other lipid-lowering medications such as ezetimibe in our dataset. Third, there is a possibility that some patients were dispensed statins but did not take it due to side effects or noncompliance; however, it is unlikely that they would keep collecting new prescriptions after the first 3 months if they did not take the statin. Third, the baseline and the beginning for the follow-up were set at 6 months, which makes the study unable to evaluate the very early postoperative period; however, it is unlikely that early postoperative mortality and these complications were related to statin use but more likely related to the initial operation. And lastly, this is a retrospective and observational study, which may still have selection bias and residual confounding despite propensity score matching and adjusted statistical models.

## CONCLUSIONS

Ongoing statin use after isolated SAVR due to aortic stenosis is associated with less composite MACE and better overall long-term survival in both adjusted statistical model and after propensity score matching. Further randomized trials are warranted to determine whether statins should be considered as secondary prevention medication after SAVR.

## Supplementary Material

ezae007_Supplementary_Data

## Data Availability

The data underlying this article were provided by national healthcare registries in Sweden and the Swedish Web System for Enhancement and Development of Evidence-based care in Heart disease Evaluated According to Recommended Therapies (SWEDEHEART). Data will be shared on reasonable request to the corresponding author if permission can be obtained from SWEDEHEART and the Swedish National Board of Health and Welfare.

## Notes

Presented at the 37th EACTS Annual Meeting on October 5, 2023, in Vienna, Austria.

## ABBREVIATIONS

aHR: adjusted hazard ratio
ICD: International Classification of Diseases
MACEs: major adverse cardiovascular events
MI: myocardial infarction
PAD: peripheral artery disease
RAS: renin–angiotensin system
SAVR: Surgical aortic valve replacement
SWEDEHEART: the Swedish Web System for Enhancement and Development of Evidence-Based Care in Heart Disease Evaluated According to Recommended Therapies registry
TAVI: Transcatheter aortic valve implantation
