# Supplementary material for: Statin treatment after surgical aortic valve replacement for aortic stenosis is associated with better long-term outcome
Source: Eur J Cardiothorac Surg. 2024 Jan 25;65(2):ezae007. doi: 10.1093/ejcts/ezae007 (PMC11462679; doi:10.1093/ejcts/ezae007)
Supplement: ezae007_Supplementary_Data [file ezae007_supplementary_data.pdf]

Supplementary material to:

Statin treatment after surgical aortic valve replacement for aortic stenosis is associated with better long-term outcome

Emily Pan[1,2], Susanne J. Nielsen[3,4], Maya Landenhed-Smith[3,4], Charlotta Törngren[3,4], Erik Björklund[3,6], Emma C. Hansson[3,4], Anders Jeppsson[3,4], Andreas Martinsson[3,5]

1. University of Turku, Turku, Finland;
2. Department of Surgery, Central Finland Hospital Nova, Jyväskylä, Finland
3. Department of Molecular and Clinical Medicine, Institute of Medicine, Sahlgrenska Academy, Universityof Gothenburg, Gothenburg, Sweden
4. Department of Cardiothoracic Surgery, Sahlgrenska University Hospital, Gothenburg, Sweden;
5. Department of Cardiology, Sahlgrenska University Hospital, Gothenburg, Sweden;
6. Department of Medicine, Southern Älvborg Hospital, Borås, Sweden

Supplementary Table S1.

ICD and ATC codes used for comorbid conditions and events, and ATC classification codes for medications.

Comorbidity	ICD9<br>1986-1996	ICD10<br>1997 -
Myocardial infarction	410	I21.0-I21.4
Diabetes	250	E10-E14
Hypertension	401-405	I10.0-I15.9
Heart failure	428	I50, I42-143.8, I11.0, I13.0, I13.2 I50, I25.5
Atrial fibrillation	427D	I48
Stroke	431-434, 436	I61.0-I64
Chronic respiratory disease	490-496	J40-J47
Renal failure	584-586	N17-N19
Malignancy	140-208	C00-C97
Congenital heart disease	745-747	Q20-Q26
Hyperlipidemia	272.0, 272.01, 272.09	E78
Peripheral artery disease	440, 443X, 444, 447	I70, I73.9, I74, I77
Left ventricular ejection fraction	Collected from SWEDEHEART	

Groups	ATC code
Beta-blockers	C07 (excluding C07AA07)
RAS inhibitors	C09
Statins	C10AA, C10BA02, C10BX06
Platelet inhibitors	B01AC
Oral anticoagulants	B01AA, B01AE, B01AF

ICD = International Classification of Diseases, ATC = Anatomical Therapeutic Classification, RAS = renin-angiotensin system

Supplementary Table S2.

Crude event rate of primary and secondary outcomes according to statin use at baseline, reported as events (95% confidence interval) per 100-person years.

Event rates per 100-person years

	Patients on statin at baseline	Patients without statin at baseline
MACE	6.8 (6.5-7.1)	5.7 (5.5-6.0)
Death	4.8 (4.6-5.1)	4.5 (4.3-4.8)
MI	0.6 (0.5-0.7)	0.4 (0.3-0.5)
Stroke	2.2 (2.1-2.4)	1.6 (1.5-1.8)
Cardiovascular mortality	2.1 (1.9-2.2)	2.0 (1.8-2.1)
New angiography	1.6 (1.5-1.7)	1.1 (1.0-1.3)
Peripheral artery disease	2.6 (2.4-2.8)	2.1 (1.9-2.2)
New valve intervention	0.5 (0.4-0.6)	0.4 (0.4-0.5)

MACE = major adverse cardiovascular event, MI = myocardial infarction

Supplementary Figure S1.

Time-updated mixed effects Cox regression analysis between ongoing statin use and primary and secondary outcomes illustrated as forest plots and presented as adjusted hazard ratio (aHR) with 95% confidence interval (CI).

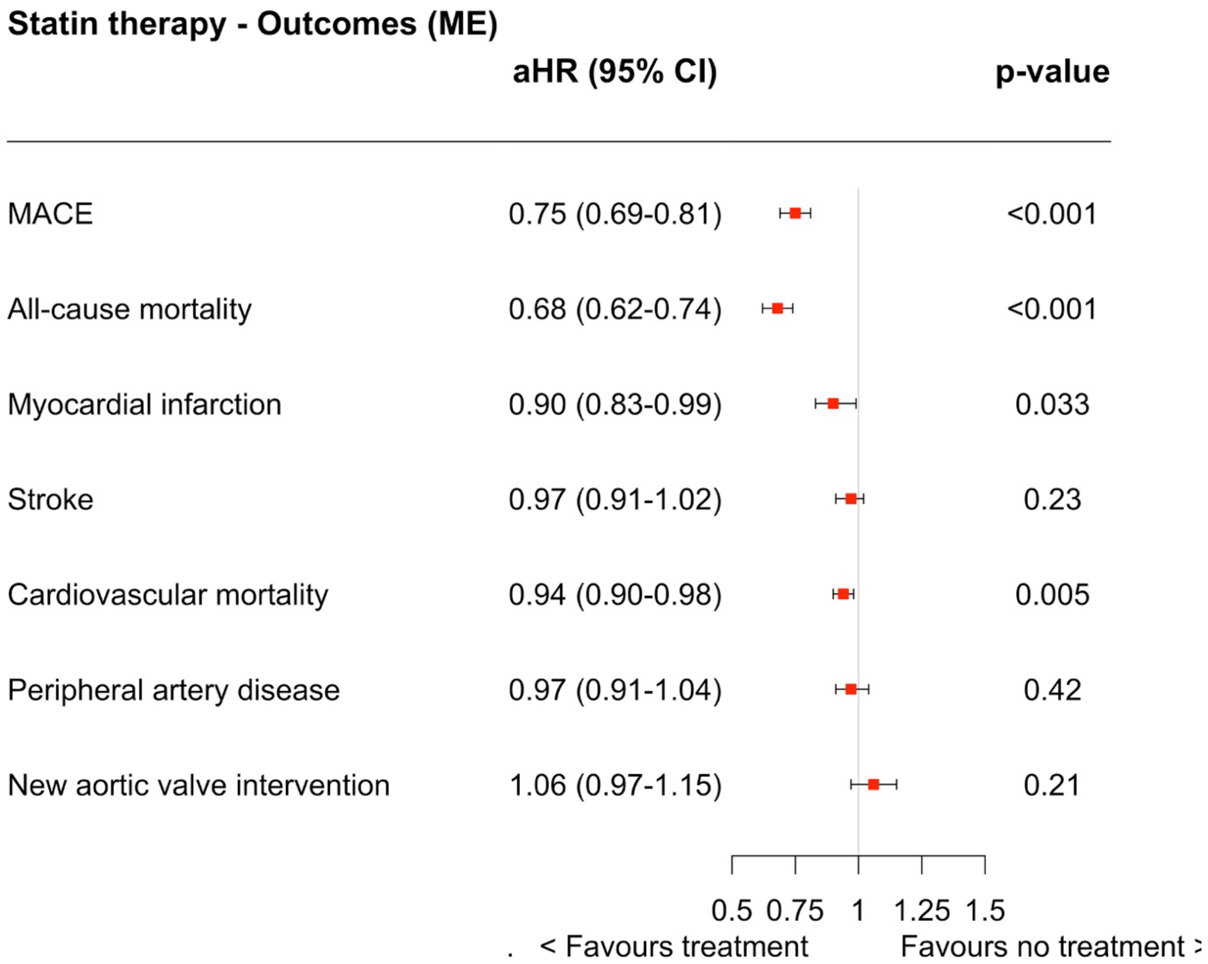

ME = mixed effects, aHR = adjusted hazard ratio, CI = confidence interval, MACE = major cardiovascular event

Supplementary Figure S2.

Mixed effects analysis showing the association between ongoing statin use and predefined subgroups, illustrated as forest plots and presented as event rate per 100 patient-year and adjusted hazard ratio (aHR) with 95% confidence interval (CI).

Statin therapy - MACE (ME)

		Number of patients (event rate per 100-py)	aHR (95% CI)	p-value for interaction
All patients			0.75 (0.69-0.81)	
Sex	Male	7,083 (5.4 (5.1-5.6))	0.72 (0.64-0.80)	0.30
	Female	4,811 (6.1 (5.8-6.4))	0.81 (0.72-0.90)	
Age	≤75 Years	7,984 (3.7 (3.5-3.9))	0.79 (0.70-0.91)	0.049
	>75 Years	3,910 (10.1 (9.7-10.6))	0.74 (0.68-0.84)	
EF <50%	Yes	2,340 (8.1 (7.6-8.5))	0.79 (0.68-0.92)	0.90
	No	9,554 (5.1 (4.9-5.3))	0.73 (0.66-0.80)	
Prior MI	Yes	947 (9.7 (8.8-10.6))	0.73 (0.59-0.91)	0.90
	No	10,947 (5.3 (5.2-5.5))	0.75 (0.69-0.82)	
Prior stroke	Yes	423 (15.4 (13.6-17.5))	0.70 (0.52-0.94)	0.78
	No	11,741 (5.4 (5.2-5.6))	0.76 (0.70-0.82)	
Peripheral artery disease	Yes	2,239 (5.7 (5.3-6.2))	0.75 (0.61-0.93)	0.89
	No	9,655 (6.3 (6.1-6.5))	0.74 (0.68-0.81)	
Hypertension	Yes	7,485 (6.6 (6.4-6.9))	0.71 (0.64-0.78)	0.087
	No	4,409 (4.3 (4.1-4.6))	0.81 (0.69-0.96)	
Diabetes	Yes	2,184 (8.4 (7.9-9.0))	0.72 (0.62-0.84)	0.73
	No	9,710 (5.1 (4.9-5.3))	0.76 (0.69-0.93)	
Hyperlipidemia	Yes	3,476 (6.8 (6.4-7.2))	0.64 (0.55-0.74)	0.042
	No	8,418 (6.0 (5.7-6.2))	0.79 (0.72-0.88)	
eGFR <60 ml/min	Yes	3,256 (10.2 (9.7-10.7))	0.85 (0.75-0.97)	0.12
	No	8,441 (4.9 (4.7-5.1))	0.70 (0.63-0.77)	
Type of prothesis	Biological	9,369 (6.7 (6.4-6.9))	0.75 (0.69-0.82)	0.76
	Mechanical	2,491 (2.5 (2.3-2.8))	0.74 (0.58-0.94)	

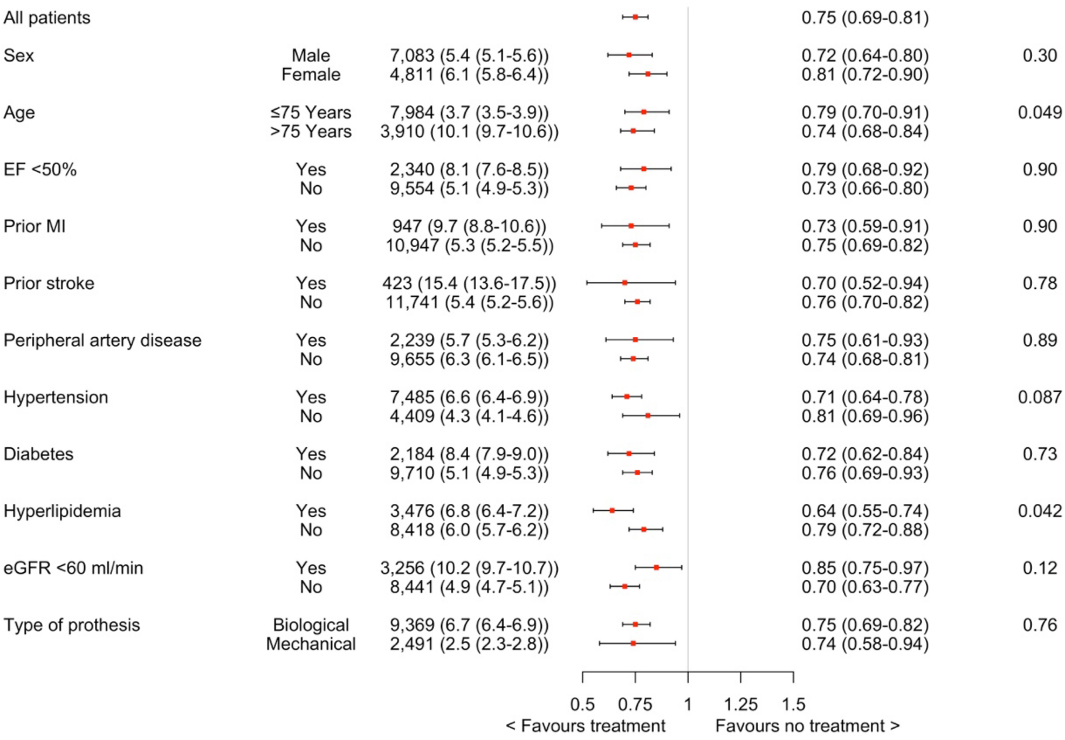

ME = mixed effects, aHR = adjusted hazard ratio, CI = confidence interval, MACE = major cardiovascular event, EF = ejection fraction, MI = myocardial infarction, eGFR = estimated glomerular filtration rate
